# Familial transmission of a body-related attentional bias – An eye-tracking study in a nonclinical sample of female adolescents and their mothers

**DOI:** 10.1371/journal.pone.0188186

**Published:** 2017-11-27

**Authors:** Anika Bauer, Silvia Schneider, Manuel Waldorf, Dirk Adolph, Silja Vocks

**Affiliations:** 1 Department of Clinical Psychology and Psychotherapy, Osnabrück University, Osnabrück, Germany; 2 Department of Clinical Child and Adolescent Psychology, Ruhr-University Bochum, Bochum, Germany; Public Library of Science, FRANCE

## Abstract

**Background:**

Previous research indicates that body image disturbance is transmitted from mother to daughter via modeling of maternal body-related behaviors and attitudes (indirect transmission) and via maternal body-related feedback (direct transmission). So far, the transmission of body-related attentional biases, which according to cognitive-behavioral theories play a prominent role in the development and maintenance of eating disorders, has not been analyzed. The current eye-tracking study applied the concepts of direct and indirect transmission to body-related attentional biases by examining body-related viewing patterns on self- and other-pictures within mother-daughter dyads.

**Methods:**

Eye movements of *N* = 82 participants (*n* = 41 healthy female adolescents, mean age 15.82 years, *SD* = 1.80, and their mothers, mean age 47.78 years, *SD* = 4.52) were recorded while looking at whole-body pictures of themselves and a control peer. Based on fixations on self-defined attractive and unattractive body areas, visual attention bias scores were calculated for mothers and daughters, representing the pattern of body-related attention allocation. Based on mothers’ fixations on their own daughter’s and the adolescent peer’s body, a second visual attention bias score was calculated, reflecting the mothers’ viewing pattern on their own daughter.

**Results:**

Analysis of variance revealed an attentional bias for self-defined unattractive body areas in adolescents. The girls’ visual attention bias score correlated significantly with their mothers’ bias score, indicating indirect transmission, and with their mothers’ second bias score, indicating direct transmission. Moreover, the girls’ bias score correlated significantly with negative body-related feedback from their mothers.

**Conclusions:**

Female adolescents show a deficit-oriented attentional bias for one’s own and a peer’s body. The correlated body-related attention patterns imply that attentional biases might be transmitted directly and indirectly from mothers to daughters. Results underline the potential relevance of maternal influences for the development of body image disturbance in girls and suggest specific family-based approaches for the prevention and treatment of eating disorders.

## Introduction

### Theoretical background

Anorexia and bulimia nervosa peak in adolescence and young adulthood [1]. In view of their serious physical and psychosocial consequences [2, 3], eating disorders are among the most severe problems experienced by adolescent girls in Western societies. Body image disturbance, a multifaceted construct encompassing weight- and body-related dissatisfaction and associated feelings, behavior and (mis)perception [4], has been shown to play a key role in the development and maintenance of such eating disorders [5].

Previous research indicated that the occurrence of body image disturbance is related to sociocultural influences (e.g., [6]). The tripartite influence model of body image and eating disturbance, a theoretical framework provided by Thompson, Heinberg, Altabe, and Tantleff-Dunn [7], suggests that parents, along with media and peers, play a crucial role in the development of body image disturbance in adolescent girls [8]. In the context of parental influence, two distinct mechanisms of impact are discussed (cp. [9, 10]). The indirect pathway of parental influence suggests–according to model learning theories [11]–that children imitate their parents’ observable body-related behavior, attitudes and comments, e. g., verbal expressions of body dissatisfaction or the use of weight loss strategies. In contrast, the direct pathway of parental influence assumes that parents’ comments about their offspring’s physical attractiveness, such as statements about shape and weight or encouragement to diet and exercise, affect the child’s body image and body-related behavior [9].

Both familial transmission paths are supported by current research, with mothers, as primary role models, apparently playing a more important role than fathers in the development of eating behavior and body dissatisfaction in girls (e.g., [12, 13]). In terms of indirect maternal transmission, associations have been found between mothers’ eating behavior and daughters’ weight control strategies [12, 14], restrained eating ([15, 16]), body dissatisfaction [17, 18] and drive for thinness [19]. In terms of direct maternal transmission, studies have shown associations between maternal body-related criticism of the daughter as well as encouragement to diet, and daughters’ body dissatisfaction [18, 20], eating problems [18], weight loss attempts [17, 21], unhealthy weight control behavior and binge eating [12].

Although many studies have investigated and confirmed the influence of direct and indirect maternal transmission on body image disturbance and associated behavior [20], certain questions remain unanswered. Previous transmission studies in the field of eating disorders were primarily based on questionnaire data about eating- and body-related behavior and attitudes. To our knowledge, the transmission of associated specific cognitive processes such as attentional biases for body- or attractiveness-related stimuli has not yet been investigated. The term “attentional bias” describes the differential, selective attentional processing of emotionally congruent material [22] and has already been investigated with regard to various forms of psychopathology [23–25]. Attentional biases to salient stimuli such as body or food cues are also considered to be highly relevant for the development and perpetuation of eating disorders [26, 27]. Selective visual attention to body-related information not only plays a prominent role in cognitive-behavioral theories of eating disorders [26, 28], but might also be a key factor in appearance-related social comparison processes as suggested in psychosocial approaches such as the tripartite influence model of body image and eating disturbance [7].

The tracking of gazes directed at body stimuli has been successfully applied in body image research as an objective measure of attention deployment [29, 30]. The measurement of eye movements allows a real-time recording of attention allocation, which enables conclusions to be drawn about underlying cognitive processes [31]. Studies differentiating between viewing patterns for one’s own and other females’ bodies found a self-serving viewing pattern in healthy females. This was characterized by stronger attention to self-defined attractive compared to unattractive body areas for one’s own body, and stronger attention to unattractive compared to attractive body areas for other females’ bodies [29, 32]. In body-dissatisfied females, the opposite gaze pattern was found, with more attention to unattractive body areas of oneself [29, 32–34] and attractive body areas of others [29, 32]. This latter viewing pattern implies upward social comparison, which in turn predicts body dissatisfaction [35]. In contrast to these findings, however, other studies demonstrated attention allocation away from “problematic” body parts, interpreted as cognitive avoidance behavior, in females high in body dissatisfaction [36], drive for thinness [37] or with diagnosed eating disorders [38]. To sum up, there is a great deal of evidence supporting differential attentional biases in females with and without body image disturbance, but findings on selective viewing patterns for salient body regions and associated social comparison processes are inconsistent.

Although body dissatisfaction and eating disorders mostly manifest in adolescence [1], to our knowledge, only two eye-tracking studies have examined selective attention to body regions in female youth [39, 40]. Horndasch et al. [39] reported biased attention to typical “problem areas” such as stomach or thighs in both girls with anorexia nervosa and healthy controls. An attentional preference for unclothed over clothed body areas was found in anorexia nervosa only, potentially reflecting the patients’ over-evaluation of shape and weight issues [39]. Svaldi et al. [40] found that the induction of a negative mood led to a stronger attentional bias for ugly body areas of one’s own body in girls with anorexia nervosa, while healthy controls showed an evenly distributed viewing pattern between ugly and beautiful body areas. This gaze behavior might reflect counter-regulatory processes in healthy girls, whereas girls with anorexia nervosa potentially reinforced their negative body schema by neglecting positive or neutral body information [40].To summarize, current research underlines the existence of a body-related attentional bias in adolescents with and without eating pathology, but does not allow conclusions about selective visual attention allocation to one’s own body compared to the body of another adolescent female. Such an investigation of body-related gaze behavior might provide insights into underlying mechanisms such as social comparison processes and associated body (dis)satisfaction [35]. Moreover, it would be especially useful to examine such behavior in adolescents without body image disturbance, as their viewing patterns might provide hints regarding protective, self-serving cognitive mechanisms (cp. [29, 40]) that prevent the development of eating pathology.

In conclusion, there is evidence to support a link between body-related attentional biases and body image disturbance in adult samples (e.g. [29]). However, so far, evidence in adolescent girls is lacking. Although the impact of maternal transmission on the development of girls’ body image disturbance and body-related behavior [9] is well documented, specific cognitive processes such as attentional biases have not yet been considered in this regard. The present study aimed to link these two etiological approaches–familial transmission and body-related attention allocation–for the first time, by transferring the concepts of indirect and direct maternal transmission to the selective visual information processing of body stimuli. For this purpose, we conducted an eye-tracking study with mother-daughter dyads, presenting each participant with body pictures of oneself and a female adolescent or adult control peer in order to analyze their gaze patterns on self-identified attractive or unattractive body areas of either stimulus. The viewing patterns were operationalized by so-called visual attention bias scores, calculated from fixation times of attractive and unattractive body areas of one’s own and the respective control body separately for mothers and daughters (cp. [32]; see also method section for detailed description). Additionally, mothers’ eye movements on their own daughter’s and the adolescent control peer’s body pictures were recorded. Based on these further assessments, a second visual attention bias score for maternal gaze behavior was calculated and its association with the bias score of the daughters was examined.

### Hypotheses

On the basis of previous research on body-related attention allocation, in a first exploratory analysis, body-related viewing patterns on one’s own and an adolescent control peer’s body in female teenagers were investigated. The main focus of this study was on the transmission of a body-related attentional bias from mothers to daughters via both transmission paths. In this regard, the first hypothesis expected a positive correlation between mothers’ and daughters’ viewing patterns on one’s own and a control peer’s body. This reflects modeling behavior in the sense of indirect transmission (i.e., is the way in which mothers inspect their own compared to another adult female’s body reflected in the way in which their daughters inspect their own body compared to another adolescent female’s body?). The second hypothesis, referring to the direct transmission path, assumed a positive correlation between the daughters’ body-related viewing patterns on one’s own and a control peer’s body and their mothers’ body-related viewing patterns on their own daughter’s body and the adolescent control peer (i.e., is the way in which mothers inspect their daughters’ bodies, as compared to how they inspect other girls’ bodies, reflected in the way in which their daughters look at themselves, as compared to how they look at other girls’ bodies?). An additional third hypothesis, also referring to the direct transmission path, expected correlations between the girls’ viewing patterns and maternal appearance-related comments about their daughters (i.e., is the way in which girls inspect their own body, as compared to another adolescent female’s body, associated with positive or negative maternal comments?). Whereas the first and second hypotheses cover rather implicit, non-verbal mechanisms of maternal transmission, the third hypothesis complements this approach by addressing the impact of observable, explicit maternal behavior on daughterly body-related attention.

## Method

### Participants

A sample of *n* = 50 female teenagers aged 13 to 18 and their mothers, thus a total of *N* = 100 participants, was recruited via press reports in regional newspapers and school presentations in Lower Saxony and North Rhine-Westphalia, Germany. For ethical reasons, general exclusion criteria were acute suicidal tendencies and self-harming behavior. For the girls as index participants, a further exclusion criterion was the presence of any mental disorder, assessed via the structured clinical interview *Diagnostic Interview for Mental Disorders in Children and Adolescents* (Kinder-DIPS [41]) based on the criteria of the Diagnostic and Statistical Manual of Mental Disorders DSM-IV [42]. In line with common practice in familial transmission research in psychopathology (cp. [43, 44]), this exclusion criterion was set for the index group only. All participants gave written informed consent, and for adolescents under the age of 18, informed consent was also obtained from their parents. Each mother-daughter dyad received an expense allowance of 30 € (approximately 33.85 US$) for their participation. According to the recommendations of Holmqvist et al. [45] regarding accuracy and precision standards of eye-tracking data, *n* = 8 mother-daughter dyads had to be excluded from the statistical analysis due to poor data quality. Additionally, *n* = 1 dyad was excluded due to incomplete questionnaires. Thus, the final sample amounted to *N* = 41 mother-daughter dyads. The analysis of the direct transmission path was conducted in a subsample of *n* = 36 dyads, as the required eye-tracking data (mothers’ viewing pattern on their daughter’s and the adolescent peer’s body) was not available for *n* = 5 dyads due to technical problems.

### Psychometric assessment

#### Eating disorder and body image pathology

Potential eating problems and body image disturbance in mothers and daughters were assessed with the Eating Disorder Examination Questionnaire (EDE-Q; [46], German version: [47]) and the Eating Disorder Inventory-2 (EDI-2; [48], German version: [49]). The EDE-Q is a self-report questionnaire which assesses the specific pathology of eating disorders in a 28-day time frame. It consists of the four subscales “Restraint”, “Weight Concern”, “Shape Concern” and “Eating Concern”, rated on a seven-point Likert scale ranging from *no days/not at all* (0) to *every day/markedly* (6). In a non-clinical sample, the EDE-Q yielded good to excellent internal consistencies ranging from α *=* .80 to α *=* .93 [50]. Internal consistencies for the current sample reached values from α = .80 to α = .91 for female adolescents and values from α = .76 to α = .88 for their mothers.

The EDI-2 [48, 49] allows for the multidimensional assessment of eating disorder pathology and related characteristics via self-report. Participants answer items of eleven subscales on a six-point rating scale from *never* (1) to *always* (6). The two body image-related subscales used for this study provide good to excellent internal consistencies for healthy female adolescents (“Body Dissatisfaction”: α = .90, “Drive for Thinness”: α = .88; [51]) and for healthy female adults (“Body Dissatisfaction”: α = .88, “Drive for thinness”: α = .88; [49]). For the current sample, internal consistencies were excellent for the adolescent females (“Body Dissatisfaction”: α = .91; “Drive for Thinness”: α = .93) and good to excellent for their mothers (“Body Dissatisfaction”: α = .90; “Drive for Thinness”: α = .84).

#### Body-related feedback

Maternal feedback about the daughter’s appearance was assessed with the Verbal Commentary on Physical Appearance Scale (VCOPAS; [52]). In its original version, the VCOPAS measures body- and appearance-related comments from an unspecific source by self-report. It consists of the three subscales “Positive Weight and Shape” (example item: “You are in great shape”), “Negative Weight and Shape” (example item: “You need to watch what you eat”) and “General Positive Appearance” (example item: “You have a beautiful smile”), rated on a five-point scale from *never* (0) to *very often* (4). For this study, the questionnaire was translated into the German language and then back-translated by a native English speaker. Discrepancies between the back-translated version and the original version were discussed and adjusted for equivalence by consensus. Furthermore, in our adaptation of the VCOPAS, the unspecific source of comments in the original version was replaced by the source “mother”. As we were interested in specific body-related feedback, only the two weight- and shape-related subscales were used in this study. Internal consistencies for the German adaptation of the VCOPAS with the source “mother”, based on a sample of non-clinical young females, were excellent for the subscale “Negative Weight and Shape”, at *α* = .92, and acceptable for the subscale “Positive Weight and Shape”, at *α* = .72 (unpublished Bachelor’s thesis). For the current sample of healthy female adolescents, Cronbach’s Alpha reached values of *α* = .84 for “Negative Weight and Shape” and *α* = .70 for “Positive Weight and Shape”.

### Procedure

The study took place in the eye-tracking laboratories of Osnabrück University and the Ruhr-University Bochum, Germany. Interested mother-daughter dyads were informed about the study procedure by telephone. As a cover story, participants were told that the eye-tracking session aimed to assess pupil dilation, which as an autonomous physical reaction cannot be altered deliberately. To avoid potential priming effects from answering questionnaires directly before the eye-tracking session, a set of questionnaires, including the EDE-Q, the EDI-2, and the VCOPAS, was sent to the participants by mail along with the study information one to two weeks before the assessment. Participants were asked to complete the questionnaires at home and bring them along to the laboratory on the day of the eye-tracking session. Mothers and daughters were invited together, but participated separately in each part of the examination. After obtaining informed consent, whole-body pictures of mother and daughter were taken by a female study assistant using a Panasonic Lumix DMC-TZ8 digital camera. The pictures were shot in front of a white screen under standardized light conditions. All participants wore the same grey underclothes (bra and briefs) and were photographed from the neck down in four standardized positions. In this study, eye-tracking data were reported only for the frontal view, as this perspective was considered to be highest in ecological validity due to its similarity to the everyday perception of oneself, e.g., when looking in a mirror. Additionally, the participants’ weight and height were measured using standardized measuring instruments. Afterwards, participants looked at their own body pictures and a peer’s body pictures consecutively on a 22” computer monitor for 6 seconds each, while spontaneous eye movements were recorded. The adolescent peer presented to the daughters was a 15-year-old girl with a body mass index (BMI) of 20.1 kg/m^2^ and the adult peer presented to the mothers was a 41-year-old woman with a BMI of 23.6 kg/m^2^. The presentation order of the pictures of oneself and the female peer was randomized by throwing a die. Before the body pictures appeared, a slide indicated whose body would be shown next (cp. [53]). Moreover, prior to stimulus onset and during the inter-stimulus intervals, a fixation cross was presented for 2 seconds. Eye-tracking data were measured by the remote contact-free eye-tracking system *RED 500*, providing an accuracy of 0.4°, a spatial resolution of 0.03° and a sampling rate of 500 Hz (SensoMotoric Instruments, Teltow, Germany). After the recording of spontaneous eye movements, the images were shown again for 6 seconds without eye-tracking, with the following instruction: “Please look closely at the pictures now, as you will be asked to evaluate them afterwards.” Subsequently, participants were asked to create an attractiveness hierarchy of twelve body areas (stomach, chest, décolleté, upper arms, lower arms, upper legs, lower legs, feet, hands, bottom, upper back, lower back) separately for their own and the peer’s body. To assess data for the examination of the direct transmission path, an additional eye-tracking trial was performed by the mothers only, who looked at their daughter’s and the adolescent peer’s body pictures. Here too, after a second photo presentation without eye-tracking, mothers’ attractiveness rankings for the body parts of their own daughter and her peer were assessed. Prior to each eye-tracking run, the accuracy, i.e., the average deviation of the recorded from the real gaze position [45], was measured. Accuracy values amounted to *M* = 0.41° (*SD* = 0.17) for the adolescents’ eye-tracking run, *M* = 0.45° (*SD* = 0.17) for the mothers’ eye-tracking run and *M* = 0.51° (*SD* = 0.24) for the mothers’ additional eye-tracking run, which meets the requirements of reliable data recording [45]. After the eye-tracking session, the structured interview Kinder-DIPS [41] was conducted with the girls. Potential mental disorders in mothers were assessed with the adult version of the interview, the *Diagnostic Interview for Mental Disorders* (DIPS; [54]). Both structured interviews were conducted by a clinical psychologist. Finally, all participants talked to the clinical psychologist about their experiences during the examination and were debriefed.

The study was approved by the ethics committee of Ruhr-University Bochum, Germany and was conducted in accordance with the ethical standards of the World Medical Association Declaration of Helsinki.

### Data analysis

The recorded eye-movement data were processed by the software program BeGaze™. Prior to the statistical analysis, data quality was checked by visual inspection of the line graphs depicting velocities during fixations and saccades, taking into account the recommendations of Holmqvist et al. [45]. Trials with insufficient data quality were excluded from the statistical examination (see description of dropouts above). In line with previous eye-tracking research (e.g., [37, 39]), a minimum fixation duration of 100 ms was defined as the lower limit for the fixations included in the statistical analyses. A bias score, which comprises the fixations on one’s own and the peer’s attractive and unattractive body parts, was calculated based on the approach of Roefs et al. [32]. Accordingly, the fixation times of the three body areas evaluated as the most attractive and unattractive for one’s own body and the peer’s body were related as follows: *(attractive self + unattractive other)–(unattractive self + attractive other)*. A positive bias score represents a self-serving gaze pattern and a negative bias score a non-self-serving gaze pattern [32]. For the supplementary eye-tracking data of the mothers viewing their daughter’s body and the adolescent peer, a second bias score was calculated following the same procedure: *(attractive areas daughter + unattractive areas adolescent peer)–(unattractive areas daughter + attractive areas adolescent peer)*. A positive bias score represents a benevolent viewing pattern on one’s daughter, a negative bias score a benevolent viewing pattern on the adolescent peer. Statistical data analysis was performed with the software program SPSS Statistics 23 (IBM; Armonk, USA). For the exploratory examination of adolescents’ body-related attention allocation, we conducted a two-way repeated measures ANOVA with the within-subject factors Body (own vs. peer’s body) and Attractiveness (self-identified attractive vs. unattractive body parts). Product-moment correlation coefficients of the daughters’ and mothers’ bias scores were calculated to examine the association of their gaze patterns on their own and the peer’s body (Hypothesis 1; indirect transmission) and to examine the relationship between the daughters’ bias score and the mothers’ additional bias score, which reflects their attentional reactions to their own daughter’s and her peer’s body (Hypothesis 2; direct transmission). For the further investigation of the direct transmission path (Hypothesis 3), again, product-moment correlation coefficients were calculated for the girls’ visual attention bias score and the subscales of the feedback questionnaire VCOPAS. A *p*-value of less than 0.05 was considered significant. For the correlation analyses, α-level was corrected using the Bonferroni-Holm procedure [55].

## Results

### Sample characteristics

Detailed information on daughters’ and mothers’ age, BMI, EDE-Q and EDI-2 scores as well as the daughters’ VCOPAS scores can be found in Table 1. The girls’ mean scores on the EDE-Q and the EDI-2 were equivalent to scores observed in a healthy female population [49, 56]. The structured clinical interview Kinder-DIPS indicated that none of the adolescents fulfilled the criteria for a current mental disorder. The mothers’ mean scores on the EDE-Q and the EDI-2 subscales were also within the healthy range [49, 57]. The structured clinical interview DIPS revealed that *n* = 3 mothers fulfilled the criteria for a mental disorder (social anxiety disorder, substance abuse, major depression with comorbid social anxiety disorder) at the time of study participation.

**Table 1:** Participants' characteristics regarding body- and eating-related symptoms.

	*Mean (Standard Deviation)*	
	Daughters (*n* = 41)	Mothers (*n* = 41)
Age (years)	15.82 (1.80)	47.78 (4.52)
Body Mass Index (kg/m^2^)	20.02 (2.48)	23.23 (3.68)
**Eating Disorder Examination-Questionnaire (EDE-Q)**		
Restraint	0.70 (1.01)	0.72 (0.98)
Eating Concern	0.60 (0.91)	0.25 (0.59)
Weight Concern	1.26 (1.24)	0.86 (1.02)
Shape Concern	1.57 (1.35)	1.20 (1.16)
**Eating Disorder Inventory-2 (EDI-2)**		
Body Dissatisfaction	3.06 (1.11)	3.09 (1.17)
Drive for Thinness	2.27 (1.19)	2.30 (0.67)
**Verbal Commentary on Physical Appearance Scale (VCOPAS)**		
Positive Weight and Shape	1.25 (0.75)	—^a^
Negative Weight and Shape	0.50 (0.56)	—^a^

Note: Average item ratings are reported.

^a^ VCOPAS scores are given for daughters only.

### Exploratory analysis of the girls’ body-related attention allocation

**Fig 1:**
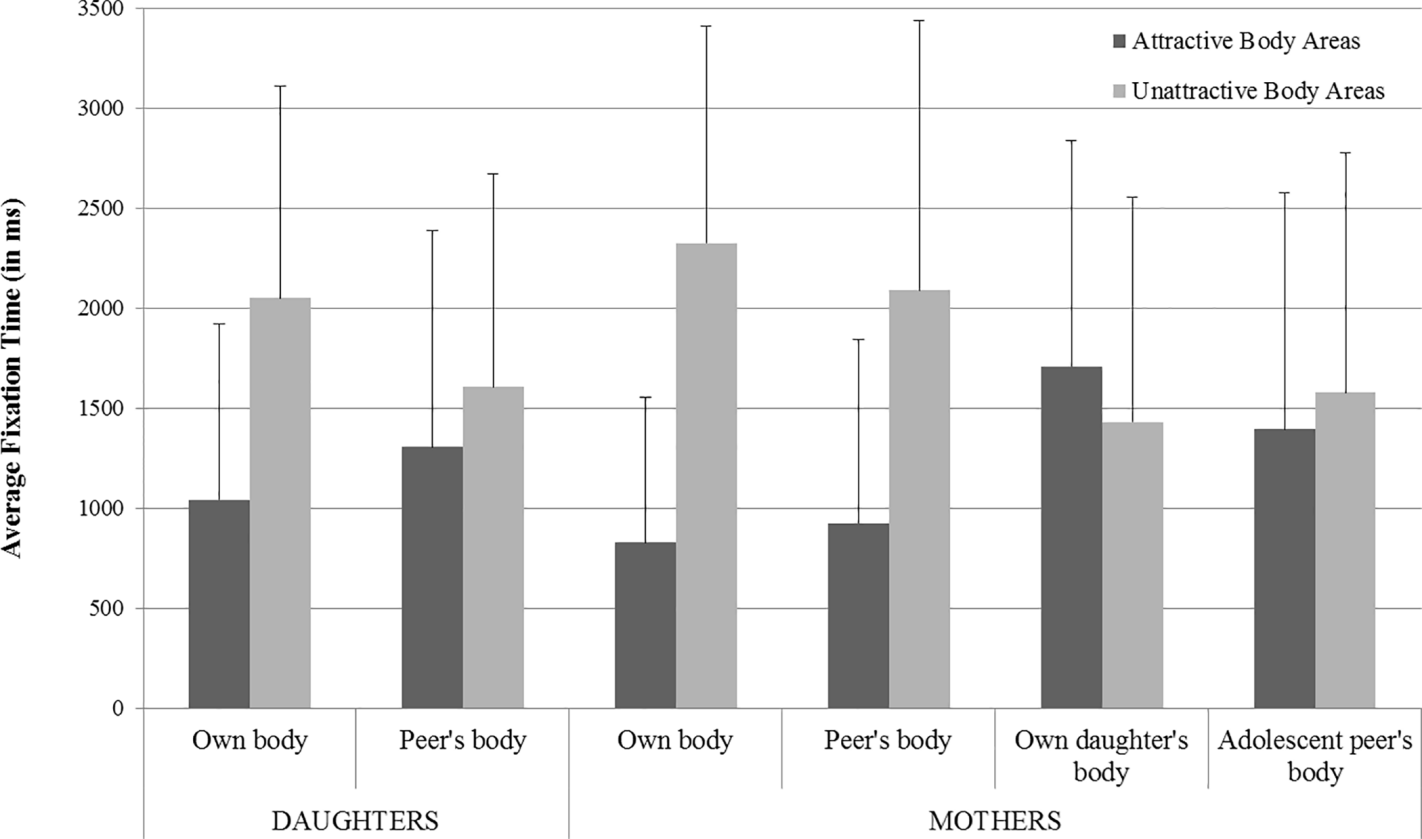
Adolescents’ and mothers’ mean fixation times on attractive and unattractive body areas.

The two-way repeated measures ANOVA with the within-subject factors Body (own vs. peer’s body) and Attractiveness (self-identified attractive vs. unattractive body parts) revealed a substantial main effect of Attractiveness, *F*(1, 40) = 12.53, *p* = .001, *η*p^2^ = 0.24, indicating significantly longer fixation times on self-defined unatractive body areas across both presented bodies (see Fig 1). According to Cohen’s conventions ([58]; see also [59]), this effect can be described as large. The main effect of Body was not significant, *F*(1, 40) = 1.02, *p* = .318, *η*p^2^ = 0.03. Moreover, the two-way Body × Attractiveness interaction failed to reach statistical significance, *F*(1, 40) = 3.13, *p* = .085, *η*p^2^ = 0.07.

### Hypotheses 1 and 2: Correlations between daughters’ and mothers’ viewing patterns

The visual attention bias scores, reflecting the attention allocation to one’s own and the control peer’s body areas (Hypothesis 1), were significantly correlated between mothers and daughters, *r* = .35; *p* = .024, with a medium effect size [58]. The descriptive data indicated a non-self-serving pattern of attention allocation in both mothers, *M* = -334.73 (*SD* = 2506.31) and daughters, *M* = -705.78 (*SD* = 2555.44).

The correlation between the girls’ visual attention bias score and their mothers’ second bias score, reflecting maternal attention allocation to their own daughter’s and her adolescent control peer’s body (Hypothesis 2), also reached statistical significance, *r* = .51; *p* = .002, with a large effect size [58]. Descriptive data showed that the mothers’ additional bias score points to a benevolent viewing pattern towards their daughters, *M* = 461.22 (*SD* = 2951.88).

### Hypothesis 3: Correlations between adolescents’ viewing patterns and maternal feedback

A significant negative correlation between the girls’ visual attention bias score and the VCOPAS scale “Negative Weight and Shape” (*M* = 0.50; *SD* = 0.56) was found, *r* = -.42, *p* = .006, with a large effect size [58]. The correlation between the girls’ visual attention bias score and the VCOPAS scale “Positive Weight and Shape” (*M* = 1.25, *SD* = 0.75) did not reach statistical significance (*r* = .10, *p* = .550).

## Discussion

The investigation of the first, exploratory research question revealed that female adolescents showed biased attention to self-defined unattractive body areas for both one’s own and the adolescent control’s body. Previous research revealed such an attentional preference for self-defined ugly body areas of oneself in body-dissatisfied women [29, 32]. However, in contrast to our results, participants from previous studies attended more strongly to attractive areas of others, suggesting upward comparison processes [29, 32]. In our sample, we found a general deficit orientation, which potentially reflects a rather self-serving attitude, as the girls attended to unattractive body areas of themselves *and* the peer’s body to a comparable extent. This means that the concentration on self-defined ugly parts of one’s own body might be “compensated” by also attending to ugly body parts of others. As focused attention on one’s own unattractive body areas results in higher levels of body dissatisfaction [60], a corresponding negative attentional focus on others might have a stabilizing effect on self-esteem and body satisfaction (cp. [36]).

However, in contrast to previous findings in healthy females [29, 32], the adolescents strongly neglected self-defined beautiful body areas. This concentration on unattractiveness may be interpreted in line with the concept of “normative discontent”, which postulates preoccupation with weight and shape and related concerns in most females [61, 62]. As puberty is characterized by profound physical changes, combined with appearance-related social pressure (e.g., [63]) and a high incidence of social comparison processes (e.g., [64]), it seems conceivable that normative discontent also plays a crucial role at a young age. Therefore, the deficit-oriented attention allocation we found can be interpreted as a reflection of the ubiquitous preoccupation with one’s physical appearance, which seems to occur even in healthy adolescents with low to moderate body dissatisfaction symptoms (see sample description in Table 1).

The body-related gaze patterns on oneself and the peer give rise to the question of the underlying neuronal correlates of visual body processing. Previous research provided discrepant findings: While some studies showed that processing of one’s own and other bodies led to differential neuronal activations in the extrastriate body area, a cortical region which responds selectively to body stimuli, in healthy females [65, 66], others did not [67, 68]. In females with eating disorders, visual attention to self- and other-physiques was shown to be associated with a lower activation of the attentional system for one’s own body [53, 69], which might be linked to typical features of body image disturbance such as body-related avoidance behavior or social comparison processes [53]. A synthesis of research on selective body-related attention on body areas and associated neuronal responses might shed light on potentially underlying neuronal mechanisms reflected in biased attention on body stimuli. Moreover, information on differential neuronal patterns in clinical and non-clinical samples might help to clarify whether eating disorders should be considered as distinct entities or represent a continuum, with healthy and body-dissatisfied individuals showing similar attentional and neuronal patterns of body processing.

The central focus of our study was the examination of associations between the observed body-related gaze behavior of adolescents and their mothers. The viewing pattern for one’s own and the control peer’s body correlated significantly between mothers and daughters. In concrete terms, the more (or less) self-serving a mother’s attention allocation to her own body is, the more (or less) self-serving is her daughter’s pattern of attention allocation to her own body. This result confirms the first hypothesis and therefore provides support for our assumption of indirect transmission processes in selective body-related attention allocation. Interpreted in the light of body-related model learning processes (e.g., [17]), the significant association between mothers’ and daughters’ viewing patterns found in the current study might tentatively be considered as resulting from daughters’ observation and internalization of their mothers’ attitudes to their own physical appearance and associated processes such as upward or downward comparison (cp. [70]).

Moreover, in line with our second hypothesis, the association between the adolescents’ viewing pattern, reflected by the visual attention bias score, and their mothers’ viewing pattern on their own daughter and the adolescent peer, reflected by the additional visual attention bias score, was significant. In other words, the more (or less) benevolent a mother’s viewing pattern on her daughter’s body was, the more (or less) benevolent was the daughter’s attention allocation to her own body. Thus, a mother’s attention allocation to her own daughter and her daughter’s peers might–as a correlate of the mother’s attitudes to her own daughter’s and other teenagers’ attractiveness–implicate social comparison processes and therefore contribute to the development and manifestation of the daughter’s body (dis)satisfaction [35].

To further analyze the direct transmission of attention allocation processes, complying with the well-documented pathway of direct influence via verbal feedback (e.g., [17]), the association between maternal comments about the daughter’s body and the daughter’s viewing pattern was examined (Hypothesis 3). In line with our assumptions, the more negative feedback a girl received from her mother, the less self-serving was her body-related viewing pattern and vice versa. These results are consistent with previous findings in the field of direct familial transmission, postulating associations between maternal comments and daughterly body dissatisfaction and associated behaviors [20]. Our study contributes the new finding that verbal criticism seems to be linked not only to daughters’ self-reported body-related behavior and attitudes, but also to specific, observable cognitive-behavioral processes such as selective visual attention allocation. Hence, this correlation further underscores our approach of transferring the concept of familial transmission to body-related attentional processes. However, a significant association was only found for negative feedback, whereas positive feedback was not related to the girls’ viewing pattern. Interpreted in the light of schema-congruent information processing [28], negative body-related feedback may be a more salient cue to female adolescents than positive feedback, as it addresses common appearance-related dissatisfaction in puberty [71]. Salient, especially negative, stimuli evoke a prioritized cognitive processing and might therefore gain higher relevance than other, less threatening information (for an overview see [22]).

To summarize, the current findings underscore previous research results providing evidence of an effect of both indirect and direct maternal transmission on daughters’ behavior and attitudes (e.g., [18]). Moreover, for the first time, this transmission approach was extended to selective attention allocation for both postulated transmission paths. Support for our interpretation that the correlated gaze patterns may be due to direct and indirect transmission processes can be found in studies in the field of familial transmission of cognitive biases in other mental disorders (e.g., [43, 72]). Several studies suggest that cognitive biases are transferable from parents to children; for example, Waters, Forrest, Peters, Bradley, and Mogg [72] found a significant association between attention biases for facial expressions in mothers with emotional disorders and attention biases in their children. Furthermore, Creswell, Schniering, and Rapee [43] reported significant correlations between anxious children and their mothers in terms of their threat interpretation. Therefore, a logical extension of this research was to analyze these mechanisms for body image disturbance.

Some limitations of the current study have to be mentioned. First, the fact that we analyzed a sample of female adolescents without any mental disorder restricts the generalizability of our results to broader populations of female youths. As mental health problems are very common in adolescence [73], our sample cannot be considered as fully representative. However, as the objectives of this study were to analyze body-related attention allocation and its transmission in healthy adolescents, we aimed to avoid potentially confounding influences such as divergent mental health conditions.

Furthermore, it should be pointed out that the correlational results of our study do not provide any evidence of a direct causal connection. The associations of mothers’ and daughters’ gaze patterns can be considered as hints for the assumed underlying learning processes, but do not allow for causal conclusions. As experimental investigations on this subject are scarcely feasible, longitudinal studies are needed to clearly identify whether specific cognitive patterns in mothers represent a predictive factor for the development of body image disturbance and associated attentional biases in children.

Finally, it is necessary to underline that the development of body image disturbance is related to different bio-psycho-social sources of influence. According to the tripartite influence model of body image and eating disturbance [7], family environment, peers and media contribute to the development of body dissatisfaction and disordered eating in adolescence, partly mediated by appearance comparison processes and the internalization of societal appearance standards [8, 74]. Besides environmental factors, genetic determinants of eating disorders are discussed [75, 76]. Furthermore, individual psychological vulnerabilities and resilience are highly relevant. For example, females with anorexia nervosa were found to differ from their healthy siblings in terms of personality traits such as need for approval, persistence and self-directedness, which may therefore act as protectors against family and environmental stressors [77]. Hence, the development of body image disturbance is a complex, multifactorial event of interacting social and individual components, of which we focused on one potential source of influence in the present study.

## Conclusion

Our study provides the first evidence supporting the existence of a selective attentional bias on individualized body stimuli in adolescent girls and the potential inter-generational transmission thereof. Due to the crucial role of attentional biases in the development and maintenance of body image disturbance [26, 28], a solid evidence basis in this field would likely lead to the implementation of new prevention and treatment approaches in body image disturbance. The systematic induction of attention to positively evaluated body areas could be helpful to prevent the development of body image disturbance in healthy adolescents (cp. [60]. Such Attentional Bias Modification training (ABM; [78]) is also thought to be a promising supplement to conventional psychotherapeutic interventions in body image and eating disorders [79].

Furthermore, the preventive application of ABM training could even be extended to the family environments of female adolescents, taking into account the role of maternal transmission in the prevention of body image disturbance in their daughters [80]. Evidence supporting the transmission of body-related attentional biases in clinical mother-daughter dyads would suggest that ABM training might be a useful, low-threshold treatment approach applicable in families with girls suffering from eating disorders.
